# Ecological and clinical evidence of the establishment of West Nile virus in a large urban area in Europe, Berlin, Germany, 2021 to 2022

**DOI:** 10.2807/1560-7917.ES.2023.28.48.2300258

**Published:** 2023-11-30

**Authors:** Claudia Ruscher, Corinna Patzina-Mehling, Julia Melchert, Selina L Graff, Sarah E McFarland, Christian Hieke, Anne Kopp, Anita Prasser, Torsten Tonn, Michael Schmidt, Caroline Isner, Christian Drosten, Dirk Werber, Victor M Corman, Sandra Junglen

**Affiliations:** 1 State Office for Health and Social Affairs (SOHSA), Berlin, Germany; 2 Institute of Virology, Charité - Universitätsmedizin Berlin, corporate member of Freie Universität Berlin, Humboldt-Universität zu Berlin and Berlin Institute of Health, Berlin, Germany; 3 German Centre for Infection Research (DZIF), partner site Charité, Berlin, Germany; 4 Experimentelle Transfusionsmedizin, Medical Faculty Carl Gustav Carus, TU Dresden and Institute for Transfusion Medicine Dresden, DRK Blutspendedienst Nord-Ost, Dresden, Germany; 5 Department of Infectious Diseases, Vivantes Auguste-Viktoria-Klinikum, Berlin, Germany; 6 Labor Berlin - Charité Vivantes GmbH, Berlin, Germany

**Keywords:** West Nile virus, mosquito, arbovirus, mosquito-borne disease, Berlin

## Abstract

**Background:**

West Nile virus (WNV), found in Berlin in birds since 2018 and humans since 2019, is a mosquito-borne virus that can manifest in humans as West Nile fever (WNF) or neuroinvasive disease (WNND). However, human WNV infections and associated disease are likely underdiagnosed.

**Aim:**

We aimed to identify and genetically characterise WNV infections in humans and mosquitoes in Berlin.

**Methods:**

We investigated acute WNV infection cases reported to the State Office for Health and Social Affairs Berlin in 2021 and analysed cerebrospinal fluid (CSF) samples from patients with encephalitis of unknown aetiology (n = 489) for the presence of WNV. Mosquitoes were trapped at identified potential exposure sites of cases and examined for WNV infection.

**Results:**

West Nile virus was isolated and sequenced from a blood donor with WNF, a symptomatic patient with WNND and a WNND case retrospectively identified from testing CSF. All cases occurred in 2021 and had no history of travel 14 days prior to symptom onset (incubation period of the disease). We detected WNV in *Culex pipiens* mosquitoes sampled at the exposure site of one case in 2021, and in 2022. Genome analyses revealed a monophyletic Berlin-specific virus clade in which two enzootic mosquito-associated variants can be delineated based on tree topology and presence of single nucleotide variants. Both variants have highly identical counterparts in human cases indicating local acquisition of infection.

**Conclusion:**

Our study provides evidence that autochthonous WNV lineage 2 infections occurred in Berlin and the virus has established an endemic maintenance cycle.

**Key public health message**

**What did you want to address in this study and why?**

West Nile virus (WNV) is transmitted by mosquitoes and causes fever or encephalitis in a fraction of infected people. Due to infrequent testing of WNV in patients, the epidemiological situation is unclear. We thus examined samples from patients with encephalitis of unknown cause for presence of WNV and performed WNV surveillance in mosquitoes in areas near acute WNV cases.

**What have we learnt from this study?**

We identified WNV infections in three cases without a history of travel in the 2 weeks before symptom onset (incubation period of the disease) and in mosquitoes collected at potential exposure sites of these cases in two consecutive years. Viral sequences derived from human cases and mosquitoes were highly similar. We show that a unique and distinct group of WNV is maintained in mosquitoes in Berlin and infected people locally.

**What are the implications of your findings for public health?**

The detection of patients presenting with West Nile fever and West Nile neuroinvasive disease together with findings of local maintenance of WNV in mosquitoes suggests that cases are underdiagnosed and we may expect numbers to increase. Enhanced surveillance combined with vector management in hot spot areas and efforts to increase awareness among physicians and veterinarians are needed to monitor and mitigate WNV cases.

## Introduction

West Nile virus (WNV) is a mosquito-borne virus (genus *Flavivirus*, family Flaviviridae) circulating between birds as amplifying hosts and ornithophilic mosquitoes of the genus *Culex* spp. as main vectors [1,2]. While humans and horses are considered non-amplifying dead-end hosts, spill over infections to humans are of public health relevance, with ca 25% of infected people developing West Nile fever (WNF) [3,4]. Severe disease progression manifesting as WNV neuroinvasive disease (WNND) is rare (< 1%). However, a case-fatality rate of ca 10% of WNND cases, with age being the most important risk factor for WNND and fatal disease outcome, has been reported [3,5]. West Nile virus transmission to humans generally occurs via mosquito bites, but contact with infectious material (e.g. blood products or organs) can also lead to WNV infection [3,6].

West Nile virus was first reported in 1937 in a febrile patient in Uganda [7]. Subsequent detections confirmed its wide geographic distribution throughout Africa, Eurasia, and recently, the Americas [8]. In Europe, several sporadic outbreaks of human WNV lineage 1 infections manifesting as spill-over infections of humans and equines occurred up to 2004, when an outbreak of WNV lineage 2 infections was reported in Hungary and Russia [9]. In subsequent years, WNV lineage 1 and 2 co-circulated in Europe leading to recurrent outbreaks in humans and equines [10]. In 2018, WNV lineage 2 caused a large outbreak throughout Europe with the highest number of human cases so far (n = 2,083) [11]. Changing climatic conditions, including exceptionally hot summer temperatures, most likely enabled the virus to spread to novel areas, including eastern Germany [11,12].

West Nile virus was detected for the first time in birds and equines in Germany during this large pan-European outbreak in 2018 [12], and the virus has recurred since then in the summer months, predominantly in eastern Germany including the city of Berlin [13-15]. West Nile virus was found one year later in mosquitoes collected near the Berlin Zoological Garden, where several WNV-positive birds were found [16]. Mosquito surveillance across a wide variety of habitats throughout Germany did not detect WNV infections in local mosquitoes before 2019 [16-18]. In addition, the German wild bird monitoring network that conducts molecular and serological surveillance for WNV in wild birds in Germany has not found WNV RNA before 2018, suggesting that the virus was first introduced during the 2018 pan-European outbreak [19]. Phylogenetic analysis showed that WNV sequences originating from Germany group together and form a so-called Eastern German clade that is most closely related to WNV sequences from Czechia [12,13,15]. The ancestor of this clade may have existed for several years in neighbouring countries before the first detection of WNV in Germany [20].

Following the identification of human cases of WNV infection in Berlin in 2021, we aimed to conduct active case finding by retrospective analysis of stored cerebrospinal fluid (CSF) samples and testing of mosquitoes caught at potential exposure sites of human index cases of WNV infection. We used virus genome analysis to identify the circulating genetic variant.

## Methods

### Epidemiological and environmental investigations

Detection of WNV infections in humans in Germany is notifiable to local public health authorities (LPHA), who in Berlin report the findings to the State Office for Health and Social Affairs Berlin (SOHSA). The case definition of a WNV infection is based on laboratory confirmation (i.e. detection of WNV nucleic acid, WNV-specific IgM or a distinct increase of WNV-IgG concentrations), and may also include symptoms of disease, defined as one or more of the following: fever, malaise, rash, muscle or joint pain and meningitis or encephalitis [21]. Since 2020, most blood donor services in Germany routinely screen blood samples obtained in the WNV transmission season (usually June to September) for the presence of WNV RNA and also report positive cases to their respective LPHA [22].

For the purpose of case investigation in 2021, LPHAs requested consent from notified cases of WNV infection for detailed interviews conducted by SOHSA. Cases were interviewed by telephone using a structured questionnaire covering data on: (i) general exposure sites, i.e. place of residence and surroundings, everyday activities; (ii) specific exposure within the 2 weeks prior to symptom onset or positive blood donation e.g. travel, outdoor activities within Berlin, contact with birds; (iii) individual risk factors for WNND, such as regular use of immunosuppressive drugs; (iv) information regarding clinical symptoms; and (v) whether household members or other persons with the same identified exposure had symptoms according to the case definition (Supplementary Annex 1, structured questionnaire).

If no travel history was reported within 2 weeks before detection of WNV infection (incubation period of the disease), mosquito trapping was initialised at potential exposure sites, i.e. where the case had spent time at dusk or dawn outdoors within those 2 weeks. Mosquitoes were trapped overnight using BG-Pro traps (Biogents, Regensburg, Germany) with a CO_2_ gas bottle at a flow rate of 1.5 mL/min and scent (BG-Lure, Biogents). In 2021, mosquitoes were collected for 1 or 2 consecutive nights with three to four traps per site. In 2022, mosquitoes were collected for 4 or 5 consecutive nights with five to six traps. A map of Berlin was created using the QGIS 3.4 software (Open Source Geospatial Foundation; http://qgis.org; 2023) implementing open source map data from Openstreetmap.org and ODIS-Berlin.de (both accessed 8 May 2023).

### Detection of West Nile virus RNA and West Nile virus-specific antibodies in human samples

Three humans were found to be WNV-positive. For Case 1, we obtained a plasma and serum sample, for Case 2, we obtained serum (days 4, 11, 13 and 27 after hospitalisation), CSF (days 4 and 13) and urine (day 6) samples and for Case 3, three CSF samples (Table 1). Further, serum was obtained from a household contact from Case 1 and from two individuals with the same exposure site as Case 2. For RNA purification, samples were processed using the MagNA Pure 96 system and the MagNA Pure 96 DNA and Viral NA Small Volume Kit (Roche Diagnostics, Mannheim, Germany) or the QIAamp Viral RNA Mini kit (Qiagen, Hilden, Germany). Purified RNA was tested for WNV either in an in-house real-time PCR (RT-PCR) set-up [23] using the Invitrogen SuperScript III One-Step RT-PCR System with Platinum Taq DNA (Thermo Fisher Scientific, Waltham, United States (US)) or the commercially available RealStar WNV RT-PCR Kit (Altona Diagnostics, Hamburg, Germany).

**Table 1:** Characteristics of human cases of West Nile virus infection in Berlin, Germany, 2021 (n = 3)

Case	Case 1	Case 2	Case 3
WNV sequence affiliation	BE_2021_01	BE_2021_02	BE_2021_03
Comment	Blood donor	Encephalitis case	Encephalitis case
Medical conditions	None	Mild adult-onset diabetes (non-insulin-dependent), arthritis	Unsolved encephalitis
Travel history (14 days before symptom onset)^a^	No travel history	No travel history	No travel history
WNV-positive sample	Blood (plasma)	Urine	CSF
Symptom onset	12 August 2021	9 August 2021	NK
Date of diagnosis	18 August 2021	26 August 2021	29 November 2021 (samples taken on 21 August, 6 September and 14 September 2021)
Sero-conversion	Yes	Yes	NK
Date of hospitalisation	NA	14 August 2021	NK
Clinical manifestations / symptoms	WNF / fatigue, fever, malaise, skin rash (arms, chest, thighs), eye pain, photosensitivity, neck stiffness	WNND / fatigue, fever, malaise, skin rash (arms, legs), eye pain, dizziness, confusion, listlessness, weakness, impaired speech, loss of appetite	WNND / NK

CSF: cerebrospinal fluid; NA: not applicable; NK: not known; WNF: West Nile fever; WNND: West Nile neuroinvasive disease.

^a^ Incubation period of the disease.

Serum samples were screened for reactive WNV IgG and IgM antibodies using a commercially available immunofluorescence test (IFT) (Anti-West Nile-Viren-IFT; Euroimmun, Luebeck, Germany). Presence of IgM antibodies was confirmed by ELISA (Anti-West Nile-Virus-ELISA (IgM), Euroimmun).

In addition, 489 CSF samples sent for broad range diagnostic testing for central nervous system pathogens between June and October 2021 were tested for the presence of WNV RNA as previously described [15].

### Mosquito identification and analysis of mosquito samples by West Nile virus real-time PCR

Trapped mosquitoes were identified on ice using a standard key [24] and stored at -80 °C. Species of WNV-positive individuals was confirmed by partial sequencing of the mosquito cytochrome c oxidase I (COI) gene as previously described [25]. PCR amplicons were treated with ExoSAP (Biotechrabbit, Berlin, Germany) according to the manufacturer’s instructions, before Sanger sequenced using Microsynth Seqlab (Microsynth, Balgach, Switzerland). Sequences were analysed using the web-tool blastn suite (National Center for Biotechnology Information, Bethesda, US).

Mosquitoes were homogenised and RNA extraction and cDNA synthesis was performed as previously described [26]. For WNV detection, RT-PCR from 2 µL cDNA was performed as described in [23].

### West Nile virus isolation and growth analysis in cell culture

West Nile virus isolated from patients, either 100 µL of serum, or 10 µL, 100 µL and 200 µL of urine, was used to inoculate Vero E6 and C6/36 cells grown to ca 80% confluency in 24-well plates. For WNV isolated from mosquitoes, cells grown in 48-well plates were inoculated with 50 µL homogenate of a WNV-positive pool or single mosquito. Each inoculum was prepared twice and either filtered with a 0.45 µm filter or treated with 0.5% Penicillin, 0.5% Streptomycin and 1% Amphotericin B. Infection was performed for 1h in a serum-free medium before a foetal calf serum-supplemented medium was added. Cells were checked daily for the appearance of cytopathic effects (CPE), and the supernatant was passaged onto fresh cells 7 days post infection. If no CPE was detected, a total of four passages were performed. Cell culture supernatants were checked for WNV replication by RT-PCR (as described above). Virus stocks were produced using Vero E6 cells and titrated with tissue culture infectious dose 50 (TCID_50_) end-point dilution assay [27].

For comparative growth analysis, Vero E6 cells grown to ca 80% confluency were infected in duplicate with WNV isolates at a multiplicity of infection (MOI) of 0.01 for 1 hour at 37 °C with 5% CO_2_. Cells were then washed once with phosphate-buffered saline (PBS) before fully supplemented Dulbecco’s Modified Eagle Medium (DMEM) was added. Immediately after infection, and consecutively every 24 hours for 5 days, 50 µl of cell culture supernatant was taken. Viral copy numbers were determined with a plasmid-based standard dilution series.

### West Nile virus neutralisation test

Serum before and after seroconversion was serially diluted in DMEM without supplements and pre-incubated with 100 TCID_50_ of WNV for 1 hour at 37 °C. Vero E6 cells grown to ca 80% confluency in 96-well plates were then infected with the serum-virus mix for 1 hour at 37 °C with 5% CO_2_, before fully supplemented DMEM was added. Cells were analysed 7 days post infection and neutralisation titres determined as lowest serum concentrations at which no CPE appeared in two replicates.

### West Nile virus whole genome sequencing and phylogenetic analyses

For complete genome sequencing, we applied two different strategies. First, we used a hemi-nested PCR amplicon strategy. Oligonucleotides were designed based on an alignment of WNV lineage 2 sequences to cover the complete WNV coding region (Supplementary Table S1, oligonucleotide sequences). For the first round of PCR, 5 µL sample RNA were amplified using the Invitrogen Superscript III one step RT-PCR system with Platinum Taq Polymerase (Thermo Fisher Scientific including 0.6 µM of each primer, 1 µL enzyme mix and additional 0.8 mM MgSO_4_, Thermo Fisher Scientific). Thermal cycling was performed at 55 °C for 15 minutes, followed by 95 °C for 3 minutes and then 45 cycles of 95 °C for 15 seconds, 56 °C for 20 seconds and 72 °C for 45 seconds, and a final elongation step of 72 °C for 2 minutes. For the second round of PCR, 1 µL first-round PCR product were amplified using the Platinum Taq Polymerase Kit (Thermo Fisher Scientific) including 2.5 µL of 10x reaction buffer provided with the kit, 1.25 µL of a 50 mM MgCl_2_ solution, 200 µM of each dNTP, 0.4 µM of each primer and 0.1 µL Platinum Taq. Thermal cycling was performed at 95 °C for 3 minutes and 45 cycles of 95 °C for 15 seconds, 56 °C for 20 seconds and 72 °C for 45 seconds followed by a 2-minute final extension step at 72 °C.

Resulting PCR products were pooled and purified with a twofold volume of KAPA Pure Beads according to the manufacturer’s instructions (Roche). For library preparation, the PCR Sequencing kit (single sample) or the PCR barcoding kit (multiple samples, Oxford Nanopore Technologies, Oxford, United Kingdom) were used and resulting libraries were sequenced on Flongle flowcells on the GridION device (Oxford Nanopore Technologies). Resulting reads were trimmed using porechop v0.2.4, mapped against reference sequence MH244512 using minimap2 v2.17-r941 and further analysed using Geneious Prime v2.2 (Biomatters, Auckland, New Zealand).

Secondly, library preparation for native Illumina sequencing was performed using the KAPA RNA Hyper Prep Kit (Roche). Five µL RNA were used for fragmentation at 85 °C for 6 minutes. After 13 amplification cycles, resulting libraries were quantified using the Qubit dsDNA HS Assay kit (Thermo Fisher Scientific) and Agilent TapeStation with the HS D1000 Kit (Agilent, Ratingen, Germany). Equimolar pooled libraries were paired-end sequenced using the NextSeq 500/550 High Output Kit (Illumina, San Diego, US).

For samples with a low-viral load or high host-background, we applied a targeted viral enrichment approach using myBaits (Daicel Arbor Biosciences, Ann Arbor, US). Here, we designed a capture bait-set using an alignment of 144 viral sequences, including 41 WNV sequences of all lineages. The final bait set comprised a total of 19,444 baits with a length of 80 nucleotides (nt) and twofold tiling density. Targeted enrichment was performed as described earlier [28].

The complete coding regions of the novel WNV and reference sequences were aligned using MAFFT version 7.450 [29]. A phylogenetic tree was constructed using the MrBayes version 3.2.6 plugin in Geneious Prime 2023 (Biomatters) with a Markov chain Monte Carlo algorithm. The GTR + I + G substitution model, as determined using jModelTest v2.1.10, was run for 3 x 10^6^ generations with reference sequence AY532665 as an outgroup.

## Results

### Identification and epidemiological investigation of West Nile virus cases

**Figure 1:**
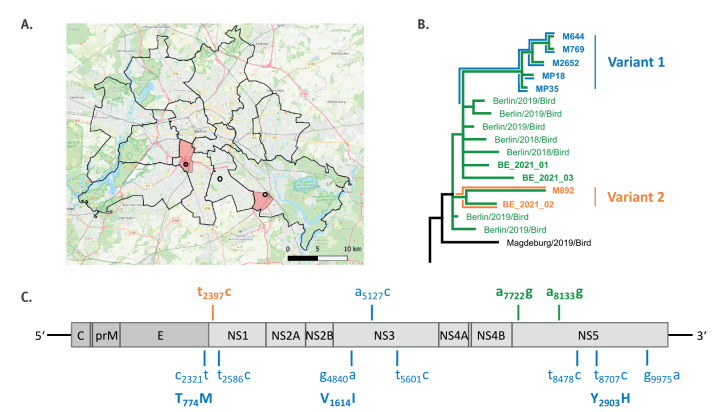
Detailed information on the detection and sequencing of West Nile virus showing (A) mosquito trapping sites in Berlin^a^, (B) phylogeny of the Berlin clade^b^ and (C) scheme of the West Nile virus genome showing the Berlin variants^c^, Berlin, Germany, 2021–2022

^a^ Mosquito trapping sites indicated by black circles. West Nile virus detection in mosquitoes indicated by red-filled circles and the urban districts in which the human cases lived are indicated by red shading.

^b^ Phylogeny of Berlin clade carrying the marker single nucleotide variants (SNVs) a7722g and a8133g (green). Variant 1 is characterised by eight additional unique SNVs, while variant 2 carries one unique SNV. The Magdeburg 2019 sequence from a bird does not include any of these SNVs.

^c^ The West Nile virus genome with the Berlin marker SNVs (green) and the unique SNVs from the Berlin variant 1 (blue) and Berlin variant 2 (orange).

Two cases of WNV infection were notified to the SOHSA in August 2021 (one female and one male with ages ranging 50–79 years). Case 1 was a blood donor from whom WNV genomic RNA was detected in plasma by a local blood donor service. They reported no pre-existing medical conditions and developed WNF shortly after the WNV-positive blood donation. Symptoms included fever, malaise, fatigue, eye pain, photosensitivity, neck stiffness and skin rash (Table 1). They recovered without lasting sequelae. Patient interviews revealed no travel history in the 14 days before blood donation. Two potential exposure sites were identified: their patient’s suburban residence; and an estate where the case spent multiple evenings outdoors (Figure 1A). One household contact with a similar potential exposure was tested for WNV-reactive antibodies, with negative result.

No detectable WNV-reactive IgM or IgG antibodies were identified in the serum sample from Case 1 taken during blood donation. However, WNV-reactive antibodies including neutralising titres of up to 1:40 were identified 19 days post blood donation, indicating seroconversion (Table 1). Attempts to isolate WNV in cell culture from Case 1 were not successful.

The second notified case, Case 2, was primarily diagnosed by the Institute of Virology at Charité – Universitätsmedizin Berlin after developing severe WNND. The patient presented symptoms of mild, non-insulin-dependent adult-onset diabetes and arthritis at admission (Table 1). No relevant travel history was reported in the 2 weeks before symptom onset (incubation period of the disease). An allotment garden plot was identified as the potential mosquito exposure site (Figure 1A). Screening of serum samples from two individuals with similar exposure at the allotment garden plot and a neighbouring plot were tested, but IFT or ELISA tests did not detect WNV-reactive IgM or IgG antibodies.

**Figure 2:**
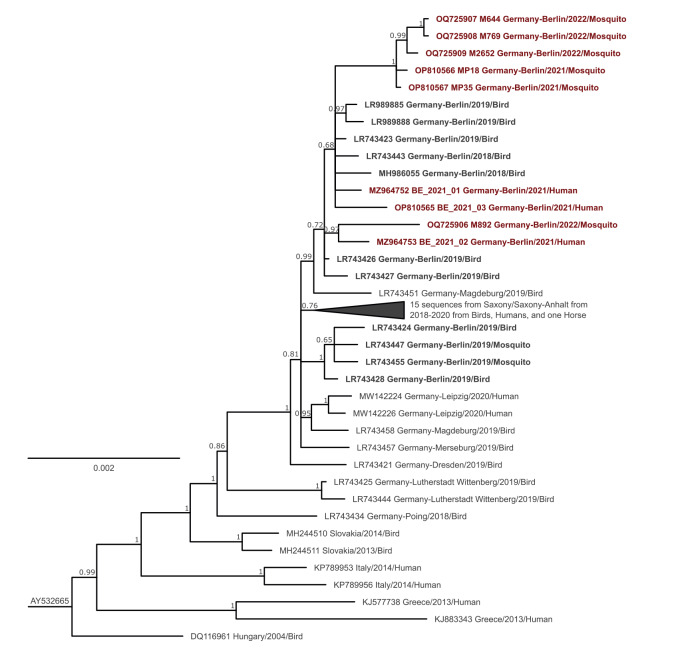
Phylogenetic relationship of the complete coding region of West Nile virus lineage 2 strains

^a^ AY532665 was used as an outgroup for rooting of the tree.

^b^ Sequences generated during this study are shown in red, sequences from Berlin are shown in bold.

^c^ Reference sequences obtained from GenBank include their accession number, country of origin, sampling year and host in the name.

West Nile virus-reactive IgM and IgG antibodies were detected in collected serum samples from Case 2 by IFT and ELISA. However, West Nile virus RNA was not detected in serum or CSF samples collected on days 4, 11 and 13 after hospitalisation, but in a urine sample taken 6 days after hospitalisation (Table 1). The virus was successfully isolated from this sample, indicating presence of infectious WNV particles in urine (Figure 2). The virus isolate was sequenced and subjected to direct serological confirmation by exposure to serum from Case 1, which neutralised cell culture infectivity up to a dilution of 1:40.

Following WNV detection in Cases 1 and 2, we retrospectively tested 489 stored CSF samples from our virological service diagnostics laboratory by WNV-specific RT-PCR. We detected viral RNA in three CSF samples taken from an already discharged patient in their thirties, who was retrospectively diagnosed with WNV infection and registered as Case 3 (Table 1). This patient had been hospitalised with an oncological disease and signs of meningoencephalitis. Due to a time lag of more than 2 months between sample acquisition and WNV detection, no interviews were possible.

### Detection of West Nile virus in mosquitoes

Mosquitoes were initially trapped at potential exposure sites of notified human cases (Figure 1A). For Case 1, sampling was performed at two exposure sites 19 and 26 days after symptom onset. For Case 2, sampling took place at one potential exposure site 30 days after symptom onset. Moreover, additional mosquito samples from the exposure site for Case 2 were recovered from an ecological field activity conducted 11 days before symptom onset. A total of 564 mosquitoes were caught, most of them at the site associated with Case 2 (n = 502), and mainly comprising mosquitoes of the *Cx. pipiens* complex (n = 478) and *Aedes Aedimorphus vexans* (n = 48) (Table 2).

**Table 2:** Information on human West Nile virus infection case-associated mosquito collection, Berlin, Germany, 2021–2022

Case	Case 1				Case 2								
Date of collection	31 Aug 2021		7 Sep 2021		29 Jul 2021		8–9 Sep 2021		25–29 Jul 2022			16–19 Aug 2022	
Site of collection	Residence		Country estate		Allotment		Allotment		Allotment			Allotment	
Mosquito species and sex	M	F	M	F	M	F	M	F	M	F	NK	M	F
*Culex (Culex) pipiens*	3	28	4	7	72	238	50	76	0	602	0	0	838
*Cx. (Culex) mimeticus*	0	0	0	0	0	0	0	0	0	0	0	1	0
*Cx.* spp.	0	3	1	15	5	4	1	0	136	874	35	120	276
*Aedes (Aedimorphus) vexans*	0	0	0	0	27	15	0	6	0	0	0	0	3
*Ae./Ochlerotatus* spp.	0	0	0	0	7	0	0	1	0	0	0	0	0
*Anopheles plumbeum*	0	0	0	0	0	0	0	0	0	0	0	0	1
*Culiseta (Culicella) fumipennis/morsitans*	0	1	0	0	0	0	0	0	0	0	0	0	0
Total	3	32	5	22	111	257	51	83	136	1,476	35	121	1,118

F: female; M: male; NK: not known.

We identified WNV in two pools of female mosquitoes (MP18 and MP35, Table 3) originating from the ecological field activity sampling pre symptom onset for Case 2. Testing of individual mosquitoes from two positive pools identified one infected female mosquito each (M132 and M310, Table 3), resulting in a WNV infection rate of 0.35% (2/564 collected at potential exposure sites in 2021. Collection dates for each site are provided in Table 2). The mosquito species was confirmed as *Cx. pipiens* complex by COI PCR and sequence analysis.

**Table 3:** Information on West Nile virus-positive mosquitoes collected in Berlin, Germany, 2021–2022 (n = 3,450)

Mosquito ID	Pool ID	Species	Sex	Collection date	Trap type + bait	Genome copy numbers per 500 µL
M132	MP18	*Cx. pipiens* complex	F	29 Jul 2021	BG Pro + CO_2_	1.04 × 10^6^
M310	MP35	*Cx. pipiens* complex	F	29 Jul 2021	BG Pro + CO_2_	1.20 × 10^7^
M640	MP65	*Cx. pipiens* complex	F	25 Jul 2022	BG Pro + CO_2_	1.41 × 10^4^
M644	MP65	*Cx. pipiens* complex	F	25 Jul 2022	BG Pro + CO_2_	3.50 × 10^8^
M769	MP78	*Cx. pipiens* complex	F	25 Jul 2022	BG Pro + CO_2_	7.42 × 10^8^
M892	MP90	*Cx. pipiens* complex	F	25 Jul 2022	BG Pro + CO_2_	2.24 × 10^7^
M2652	MP266	*Cx. pipiens* complex	F	18 Aug 2022	BG Pro + CO_2_	7.58 × 10^8^

F: female.

To test for WNV persistence, mosquitoes were sampled in the subsequent summer in the vicinity of the WNV-positive exposure site (neighbouring allotment plot, r < 300 m). This sampling yielded 1,647 mosquitoes in July 2022 and 1,239 in August 2022, which were almost exclusively mosquitoes of the *Cx. pipiens* complex (Table 2). West Nile virus was detected in five individual female *Cx. pipiens* mosquitoes, albeit in one with low genome copy numbers, resulting in an infection rate of 0.17% (5/2,886) (Table 3). The latter detection was confirmed by two independent quantitative RT-PCR assays.

### Genetic analysis of mosquito-associated West Nile virus lineages

The complete WNV coding regions from all three human cases, two positive mosquito pools from 2021 (MP18 and MP35) and four of the five positive individual mosquitoes from 2022 (M644, M769, M892 and M2652) were sequenced using high-throughput sequencing. All available complete genome sequences from Germany were included in the phylogeny, placing the recent Berlin sequences in a monophyletic clade (Figure 2). Four sequences detected in birds and mosquitoes in Berlin in 2019 fell outside this clade. Sequences within the Berlin monophyletic clade contained the two synonymous and unique single nt variants (SNVs) a_7722_g and a_8133_g (Figure 1B and C).

Five mosquito-derived sequences (MP18, MP35, M644, M769 and M2652) from 2021 and 2022 established their own sub-clade within the Berlin monophyletic clade, designated variant 1 in Figure 1B. The sequences showed high nt identities with less than four nt substitutions, and two sequences were identical (M644 and M769). Eight unique and common SNVs were identified in these five genomes, three of them non-synonymous (T_774_M in the E protein, V_1614_I in the NS3 protein and Y_2903_H in the NS5 protein; Figure 1B and C).

The WNV sequence obtained from mosquito M892 also collected at the same allotment plot in 2022 showed up to 26 SNVs from variant 1. M892 was a part of the Berlin monophyletic clade but did not cluster within variant 1, indicating that at least two different virus subvariants were infecting mosquitoes in 2022 (Figure 1B and C; Figure 2).

### Relationship between mosquito- and human-derived West Nile virus sequences

Variant 1 mosquito-associated sequences showed nt identities between 99.803% and 99.884% to the sequences from Cases 1 and 3, which fell within the Berlin monophyletic clade (Figure 2).

Viral sequences from variant 2, mosquito M892 (detected 2022) and human Case 2, were 99.845% identical and shared the silent and unique SNV t_2397_c (Figure 1B and C, variant 2). The sequences were placed as sister branches with significant bootstrap support and no other sequence directly related (Figure 2). These data indicate direct acquisition and also suggest that WNV variant 2 was already present in mosquitoes in 2021 but not detected due to insufficient sample size (we sampled at the exposure site of Case 2 11 days before symptom onset and detected only variant 1).

**Figure 3:**
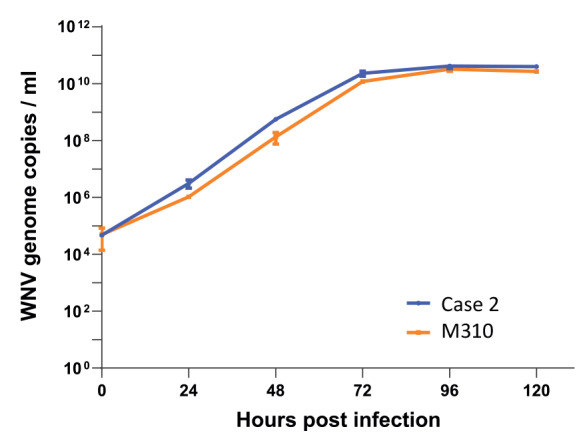
Comparative growth curves of two West Nile virus isolates from a human and a mosquito on a primate cell line, Berlin, Germany, 2021–2022

In a preliminary attempt to test for phenotypic differences between variants 1 and 2, WNV was isolated in cell cultures from mosquito M310 (pool MP35) and compared with the isolate obtained from Case 2. Growth analysis of the mosquito isolate M310 (variant 1) and the isolate from Case 2 (variant 2) did not show significantly different replication kinetics in Vero E6 cells (Figure 3).

WNV: West Nile virus.

^a^ Error bars depict the standard deviation of two replicates.

^b^ Vero E6 cells were infected in duplicates with WNV isolates BE_2021_02 (isolate from Case 2) and M310 (isolate from single positive mosquito from pool MP35) at an MOI of 0.01. Samples were taken every 24 hours for genome copy determination by RT-PCR.

## Discussion

The data presented here provide strong evidence for local establishment and transmission of WNV in Berlin. We recorded symptomatic WNV infections in three residents with no history of travel in the 14 days before symptom onset (incubation period of the disease) and detect infected mosquitoes at a potential exposure site in Berlin, and again at the same site one year later. Phylogenetic analyses based on complete genome sequences obtained from human cases and mosquitoes show that the sequences are closely related and group together, with WNV sequences previously detected in birds and humans only in the Berlin area. Importantly, WNV sequences detected in mosquitoes collected at the most likely exposure site of a human case in 2021 and 2022 carried five synonymous and three non-synonymous SNVs, suggesting that this variant has overwintered and is locally enzootic since at least 2 years.

Previous studies have detected WNV in hibernating mosquitoes collected in north-eastern Germany from mid-December 2020 to early March 2021 [30]. Human WNV infections have occurred in north-eastern Germany since 2019 and are considered to be mainly autochthonous [13-15]. Numbers of probable autochthonous human WNV infections in Germany have increased to 23 mostly symptomatic cases, including one fatality in 2020, five infections in 2021, and 19 infections in 2022, of which five were from Berlin [31]. Classification of cases as autochthonous in the studies from north-eastern Germany was based on epidemiologic assessment (as there was no history of travel to known endemic areas) and close phylogenetic relationship to WNV sequences previously detected in the same region. However, the virus has not previously been detected in humans and mosquitoes at the same time and place in Germany. According to the present results, Berlin represents one of the northernmost areas with established WNV circulation in Europe. The only other northern European area reporting infected mosquitoes and humans with compatible virus strains is the Netherlands [32].

*Cx. pipiens* mosquitoes show a high ecological plasticity and infest urban environments. They accounted for the vast majority of mosquitoes in our sample (98%). In vivo experiments have shown that indigenous *Cx. pipiens* (biotypes *pipiens* and *molestus*) showed high WNV transmission efficiencies, even at low temperatures of 18 °C [33], suggesting that even temperate climates in central and northern Europe may support WNV circulation. It has been shown that an increase in temperature strongly impacts vector competence, for example via a shortening of the extrinsic incubation period in *Cx.* spp. mosquitoes and via higher transmission rates caused by increased WNV replication in salivary glands (reviewed in [34]). West Nile virus transmission in mosquitoes has been shown to increase nonlinearly with increasing extrinsic incubation temperature, suggesting that even a small temperature increase has the potential to substantially increase WNV transmission [35]. In addition, lower precipitation is associated with increased mosquito infection and human incidence [36,37]. Thus, an increase in hot and prolonged summers in Germany combined with increasing drought in Berlin and eastern Germany is expected to increase WNV transmission and infections including WNND, as observed recently in Italy and Spain [38,39]. As WNND accounts for ca 1% of infections, the number of undetected mild or asymptomatic infections are likely to be much higher [40].

The endemic seasonal circulation of WNV lineage 2 in Berlin is most likely being maintained via WNV overwintering in infected adult local mosquitoes. This is supported by the detection of closely related WNV sequences in mosquitoes sampled at the same site in two consecutive summer seasons, with several mosquito generations in between. Specifically, variant 1 was found in the mosquito population of the allotment plot in 2021 and 2022. Variant 2 was detected in a case in 2021 and in a mosquito in 2022. Both findings collectively indicate the cocirculation of different viruses and the overwintering of both variants at the same site.

It is difficult to define the time at which WNV was locally established. Since 2016, surveillance of WNV circulation in Berlin has involved testing mosquitoes caught in the zoo in the eastern part of Berlin for the presence of WNV, but yielded no positive findings until 2019 [41]. The first detected WNV sequences in these mosquitoes were most closely related to WNV sequences found in diseased birds in eastern Germany in 2018, not pertaining to the Berlin-specific monophyletic clade described here [16]. Overall, this suggests that the WNV variants reported in the present study may have been introduced to Berlin after 2018.

The rather small mosquito sample collected for this study is a limitation. A more exhaustive sampling approach would be necessary to draw conclusions on the geographic spread and genetic diversity of WNV in Berlin. Also, while preliminary virus replication studies in Vero cells yielded no significant phenotypic changes, more detailed studies including different WNV variants and cell lines derived from different vectors, hosts and organs are required. Another limitation is that we cannot definitely conclude that the human cases in this study were infected at their presumed exposure sites as WNV-infected birds have been found in different parts of Berlin since the first emergence in 2018 [42]. However, sequences from Case 2 and mosquito M892 are not only spatiotemporally close, but uniquely genetically related.

## Conclusions

An endemic transmission cycle of West Nile virus has established in Berlin and the virus is likely to overwinter in diapausing *Cx.* spp. mosquitoes. West Nile virus infections and associated symptoms of disease, including neuroinvasive WNV infections, are likely to increase in Berlin in the upcoming years. West Nile virus surveillance in Germany relies on the detection of WNV in clinically apparent cases (usually presenting with symptoms of WNND) as well as the detection of infections by blood donor services and their notification to LPHAs. Further detection of WNV infections in patients depends on the awareness of physicians to consider WNV as causative agent when patients are presenting with febrile infections, skin rashes, muscle or joint pain. As demonstrated in the present study, systematic WNV screening in cases compatible with WNND enables the retrospective detection of cases that otherwise would remain undiagnosed. However, WNND accounts for ca 1% of infections, the number of undetected mild or asymptomatic infections being much higher. There is therefore a need for heightened awareness among physicians and veterinarians combined with complementary active surveillance concepts including surveillance in sentinel animals (such as mosquitoes, birds and equines). Also, epidemiological investigation of autochthonous WNV cases should be accompanied by surveillance of mosquito populations and vector control measures in potential hotspot areas.

## Supplementary Data

191000 Supplementary Material
