# Supplementary material for: Ecological and clinical evidence of the establishment of West Nile virus in a large urban area in Europe, Berlin, Germany, 2021 to 2022
Source: Euro Surveill. 2023 Nov 30;28(48):2300258. doi: 10.2807/1560-7917.ES.2023.28.48.2300258 (PMC10690859; doi:10.2807/1560-7917.ES.2023.28.48.2300258)
Supplement: Supplementary Material [file 23-00258_JUNGLEN_Supplementary_Material.pdf]

Supplementary material

This supplementary material is hosted by Eurosurveillance as supporting information alongside the article "Ecological and clinical evidence of the establishment of West Nile virus in a large urban area in Europe, Berlin, Germany, 2021 to 2022", on behalf of the authors, who remain responsible for the accurarcy and appropriateness of the content. The same standards for ethics, copyright, attributions and permissions as for the article apply. Supplements are not edited by Eurosurveillance and the journal is not responsible for the maintenance of any links or email addresses provided therein.

Supplementary Table 1: Oligonucleotides used for amplification and sequencing of overlapping WNV genome fragments. Used in 1st and/or 2nd round of RT-PCR as indicated.

Name	Sequence 5'-3'	Use
WNV_frag01_F_76	CAA TTA ACA CAG TGC GAG CTG T	1st round and 2nd round
WNV_frag01_Rnest_826	AGT ATC CAT GAT TCTGTC TTC ACC AG	2nd round
WNV_frag01_R_877	ATC CAT CCA ATG ACC GCT GC	1st round
WNV_frag02_F_673	GAA GAC ATT GAC TGC TGG TGC A	1st round and 2nd round
WNV_frag02_Fnest_781	ACA TGG AGA AAG TAC ACT GGC CA	2nd round
WNV_frag02_R_1239	CCA CAA CGC CTT GCT TGC A	1st round
WNV_frag03_F_1120	AAT GAT GAA CAT GGA AGC AGC CA	1st round and 2nd round
WNV_frag03_Fnest_1165	GTT ACC TAG CTT CGG TCA GYG	2nd round
WNV_frag03_R_1768	GAG AAC TCA ACA GGA ATC GCT CC	1st round
WNV_frag04_F_1603	TGG TGC GAA GTC CTT CCT GG	1st round and 2nd round
WNV_frag04_Fnest_1711	GTT TGA AGA ACC TCA TGC CAC CA	2nd round
WNV_frag04_R_2227	AAT CCC AAG CAG TGT CTC CAA G	1st round
WNV_frag05_F_2086	AGC CAA CTC GAA GGT CTT GAT TG	1st round and 2nd round
WNV_frag05_Fnest_2121	CCG TTT GGT GAC TCT TAC ATC GTG	2nd round
WNV_frag05_R_2668	CTC ATC CTT AAT GGC TTC CCA CAT	1st round
WNV_frag06_F_2506	ATT GAT ATT GGC AGG CAA GAG C	1st round and 2nd round
WNV_frag06_Fnest_2575	CTG GAT GGA TCG TTA CAA GTT CTA CC	2nd round
WNV_frag06_R_3124	GGC CAG GTG CAT GAT TTG ACT TC	1st round
WNV_frag07_F_2971	GGA TTG ACA AGC ACT CGC ATG T	1st round and 2nd round
WNV_frag07_Fnest_3073	GCA TAG TGA TCT GTC GTA CTG GA	2nd round
WNV_frag07_R_3670	CGC AGG ACA TCA GTG TAC GTA AT	1st round

WNV_frag08_F_3549	CAA CGC CGA CAT GAT TGA TCC T	1st round and 2nd round
WNV_frag08_Fnest_3608	GAG GTC CTT CGC AAG AGG TG	2nd round
WNV_frag08_R_4315	AAC ATR AGT CCG GCR ATG GTC AT	1st round
WNV_frag09_F_4141	GGC TTG CCT CAT CTG CCT AG	1st round and 2nd round
WNV_frag09_Fnest_4183	AGT GTT CAA TCC AAT GAT ACT TGC AG	2nd round
WNV_frag09_R_4885	CGA CAA TCA TTT GGA CCT CAT CAT G	1st round
WNV_frag10_F_4738	TGG AGC CGG AGT GAT GGT AG	1st round and 2nd round
WNV_frag10_Fnest_4810	GAG TGG TGA AGG ACG TCT GGA TC	2nd round
WNV_frag10_R_5362	ACG ATC TCA TTT CCA CTG TGC TC	1st round
WNV_frag11_F_5251	CCA AAT CAT CAA GGA GGC CAT CA	1st round and 2nd round
WNV_frag11_Fnest_5314	TGC TGC TGA GAT GTC TGA GG	2nd round
WNV_frag11_R_6019	GCC CTC CAT ARC AAT ACT CAT CAC C	1st round
WNV_frag12_F_5794	GAA CAG AAA GTC CTA TGA GAC AGA GT	1st round
WNV_frag12_Fnest_5887	AGG CAA GCA GAG TGA TCG AYA	2nd round
WNV_frag12_Rnest_6484	CAT TCT CCC GAG CAC CTC AAC	2nd round
WNV_frag12_R_6535	CCA CAT ACA TCG TGT CCA ACG C	1st round
WNV_frag13_F_6334	CCT CGA ACC AAC ACG ATT CTT G	1st round and 2nd round
WNV_frag13_Fnest_6412	AGG TGG GCG GAT GCY AGA G	2nd round
WNV_frag13_R_7138	GAA CAA YGC ACT GGC TTG GAC	1st round
WNV_frag14_F_6952	GGC TGG ACA ARA CCA AGA ATG ACA	1st round and 2nd round
WNV_frag14_Fnest_7102	CCT TTG CTG AAA CAT CTA ATC ACG T	2nd round
WNV_frag14_R_7726	CCT TCG TCA TGT GGT TGA GTC T	1st round
WNV_frag15_F_7636	GAT GGC TCT CGT GTC TMT CCA	1st round and 2nd round
WNV_frag15_Rnest_8335	CGTGGCTCACCCARTACATCTC	2nd round
WNV_frag15_R_8464	TGC CCG CGT TCC ACT TCC	1st round
WNV_frag16_F_8155	CGT CAA GTG CCG AGG TWG AAG	1st round and 2nd round
WNV_frag16_Rnest_8596	TTCATAGCTTCCGTGRTAGTTCCA	2nd round
WNV_frag16_R_8686	ATCGTGGTCACATTGGTGATRGTGTC	1st round
WNV_frag17_F_8401	GTG AAC ATG ACA AGC CAG GTG C	1st round and 2nd round
WNV_frag17_Fnest_8470	AGG AAG ATG TCA ACT TGG GAA GTG	2nd round
WNV_frag17_R_8983	GCT CCT CAT CCA CCA TYT CCC A	1st round
WNV_frag18_F_8938	AGG AGC GAT GTT TGA AGA ACA GA	1st round and 2nd round
WNV_frag18_Rnest_9397	GAC TTT GTG TCG GTA KGT GAG CTC	2nd round
WNV_frag18_R_9511	TTG GTG AAG GTG TTY AGG GCG TA	1st round

WNV_frag19_F_9205	GGG TAG GAA GAA CTC AGG AGG AG	1st round and 2nd round
WNV_frag19_Fnest_9313	CAG GCT GGG ACA CMC GCA	2nd round
WNV_frag19_R_9976	ATT AAA GCA GCA GCC ACA TCT G	1st round
WNV_frag20_F_9739	CTT CGC CAC ATC ACT MCA CTT CC	1st round and 2nd round
WNV_frag20_Fnest_9814	GGT GGT ATG AYT GGC AGC AGG	2nd round
WNV_frag20_R_10409	CTA CAA CTA TAC AAC TAC TCA ATA CAG CT	1st round

Supplementary Annex 1: Questionnaire

Fill out before interview:	
Date of Interview:	
Interviewer:	
Case ID Number:	
Time frame before symptom onset, or, where applicable, blood donation. (Calculation: date of symptom onset/blood donation – 14 days)	From: To:
Fill out only if case notified through blood donation:	
Date of blood donation:	
Name of blood donation service:	
Contact information (address, phone) of blood donation service:	

DEMOGRAPHICS	
Date of Birth:	
Sex:	

Blood Donation		
1. Do you donate blood regularly or have you donated blood in the last four weeks?		
Yes		
No		
Not sure / No answer		
	If yes, when and where did you last donate blood before your donation that tested positive?	
	Date:	
	Location:	

Exposure: General (without a specified time frame)
2. What is your current address of residence?

3. **Where is your house situated?**		
Apartment building within the city		
Apartment building on the outskirts of the city or in a rural/green setting.		
Single house within the city		
Single house on the outskirts of the city or in a rural/green setting		
4. **Are the following in the immediate vicinity of your place of residence?**		
Gardens/Allotments		
Courtyard		
Water sources (e.g., bird fountain, fish pond, etc.)		
Ponds, Lakes, other bodies of water		
Parks/Green spaces		
5. **Can you describe a typical day for you? For example, where you spend your time, your mode of transport and routes?**		

Exposures (in the 14 days before symptom onset or blood donation)
6. Where is your place of work?
What do you do for a living?
7. What hobbies have you undertaken?
What?
Where?
When (On what dates)?
8. Do you own an allotment or have you spent time in a garden?
Where (Address)?
When (On what dates)?
9. Have you visited a physician?
Where?
When (On what dates)?

10. Have you visited family or friends?			
Where?			
When (On what dates)?			
11. Have you travelled outside of Berlin?			
Yes		No	
When (Time frame with beginning and end date)?			
Where?			

12. Have you visited the following?		
Zoo/petting zoo		
Bird breeder/Pet store with birds		
Farm/Riding farm		
Lake/Pond		
Cemetery		
Park		
Other:		

13. Have you spent longer than an hour outdoors?	
Where:	
When (On what dates):	

14. Have you had contact with animals, especially birds?		
Yes		
When?		
Where?		
What animals?		
No		
Not sure / No answer		

**15. Have you seen or come into contact with any sick/injured or dead birds?**		
Yes		
When?		
Where?		
Do you know what type of bird?		
No		
Not sure / No answer		
**16. Can you recall a situation in which you encountered mosquitoes or in which you noticed mosquitoes?**		
Yes		
When (On what dates)?		
Where?		
No		
Not sure / No answer		
**17. Do you recall any insect bites; in particular, mosquito bites?**		
Yes		
When (On what dates)?		
Where?		
No		
Not sure / No answer		

Patient medical history / Risk factors		
18. **Do you have any of the following medical conditions or does any of the following apply to you?**		
Diabetes		
Hypertension		
Renal insufficiency		
Liver disease		
Haematological disorder		
Organ transplant		
Cancer *(in particular, brain tumour)*		
19. **Do you regularly take medication immunosuppressive medications?** e.g.: Cortisone, Prednisolone, Cyclosporine A, Cystostatics (e.g., Azathioprine)		
Yes		
No		
Not sure / No answer		
20. **Have you had any of the following symptoms?**		
Fever		
Fatigue		
Headache		
Malaise / Feeling unwell		
Muscle pain		
Vomiting		
Skin rash		
Ocular pain		
Encephalitis/Meningitis		
Paralysis		
Other		
21. **When did your symptoms begin?**		
Date:		
22. **Have you been hospitalised?**		
Yes		
When (Time frame with beginning and end date)?		
No		
Not sure / No answer		
23. **Have people with which you have had close contact (e.g., in the same household) had an illness (especially a febrile illness) in the past four weeks?**		
Yes		
When (Time frame with beginning and end date)?		
May we contact this person(s)?		
No		
Not sure / No answer		
